# Predominance of PVL-negative community-associated methicillin-resistant *Staphylococcus aureus* sequence type 8 in newly diagnosed HIV-infected adults, Tanzania

**DOI:** 10.1007/s10096-021-04160-2

**Published:** 2021-02-14

**Authors:** Joel Manyahi, Sabrina J. Moyo, Said Aboud, Nina Langeland, Bjørn Blomberg

**Affiliations:** 1 Department of Clinical Science, University of Bergen, Bergen, Norway; 2 National Advisory Unit for Tropical Infectious Diseases, Department of Medicine, Haukeland University Hospital, Bergen, Norway; 3 Department of Microbiology and Immunology, Muhimbili University of Health and Allied Sciences, MUHAS, P.O. Box 65001, Dar es Salaam, Tanzania

**Keywords:** Panton-Valentine leukocidin-negative, Sequence type 8 (ST8), Methicillin-resistant *Staphylococcus aureus* (MRSA), Human immunodficiency virus, Community, Tanzania

## Abstract

Difficult-to-treat infections caused by methicillin-resistant *Staphylococcus aureus* (MRSA) are of concern in people living with HIV infection as they are more vulnerable to infection. We aimed to identify molecular characteristics of MRSA colonizing newly diagnosed HIV-infected adults in Tanzania. Individuals newly diagnosed with HIV infection were recruited in Dar es Salaam, Tanzania, from April 2017 to May 2018, as part of the randomized clinical trial CoTrimResist (ClinicalTrials.gov identifier: NCT03087890). Nasal/nasopharyngeal isolates of *Staphylococcus aureus* were susceptibility tested by disk diffusion method, and cefoxitin-resistant isolates were characterized by short-reads whole genome sequencing. Four percent (22/537) of patients carried MRSA in the nose/nasopharynx. MRSA isolates were frequently resistant towards gentamicin (95%), ciprofloxacin (91%), and erythromycin (82%) but less often towards trimethoprim-sulfamethoxazole (9%). Seventy-three percent had inducible clindamycin resistance. Erythromycin-resistant isolates harbored *ermC* (15/18) and *LmrS* (3/18) resistance genes. Ciprofloxacin resistance was mediated by mutations of the quinolone resistance-determining region (QRDR) sequence in the *gyrA* (S84L) and *parC* (S80Y) genes. All isolates belonged to the CC8 and ST8-SCC*mec*IV MRSA clone. Ninety-five percent of the MRSA isolates were *spa*-type t1476, and one exhibited *spa*-type t064. All isolates were negative for Panton-Valentine leucocidin (PVL) and arginine catabolic mobile element (ACME) type 1. All ST8-SCC*mec*IV-*spa*-t1476 MRSA clones from Tanzania were unrelated to the globally successful USA300 clone. Carriage of ST8 MRSA (non-USA300) was common among newly diagnosed HIV-infected adults in Tanzania. Frequent co-resistance to non-beta lactam antibiotics limits therapeutic options when infection occurs.

## Background

Methicillin-resistant *Staphylococcus aureus* (MRSA) infections are difficult to treat and MRSA-bacteremia is associated with increased risk of fatal outcome [1]. Nasal colonization with MRSA is a risk factor for invasive disease [2] and a particular threat to HIV-infected individuals who are more susceptible to severe bacterial disease, including staphylococcal infections [3, 4]. Therefore, spread of community-associated methicillin-resistant *Staphylococcus aureus* (CA-MRSA) is of obvious concern [5] and particularly so in communities with large vulnerable population of HIV-infected people.

Understanding community transmission dynamics and spread of emerging CA-MRSA clonal populations is critical for the control and prevention of MRSA infection in HIV disease, and sequence typing can help shed light on the molecular epidemiology of circulating CA-MRSA population structures [6, 7].

Despite the fact that nasal colonization with *Staphylococcus aureus*/MRSA is a risk factor for invasive infection in HIV-infected individuals, there are few countries in an African setting, Botswana, Ethiopia, Nigeria, and South Africa, which have reported carriage of MRSA among HIV-infected individuals [8–11]. These reports have focused on prevalence and risk factors of *Staphylococcus aureus* and MRSA using either conventional or PCR-based methods for detection and characterization of MRSA isolates. In Tanzania, no study has reported carriage of MRSA among HIV-infected individuals, and there is only one study from the Northern part of Tanzania which has described MRSA clone populations in clinical isolates, using whole genome sequencing technology [12]. Therefore, in Tanzania and many resource-limited countries in Africa, detailed information on the CA-MRSA population structure among HIV-infected individuals is scarce. As a result, measures for prevention and control of MRSA spread are insufficiently implemented in most health care facilities. This study used whole genome sequencing approach to provide data on nasal/nasopharyngeal carriage of MRSA among newly diagnosed HIV-infected adults in a community setting in Tanzania. Additionally, we report sequence types, virulence genes, and phylogenetic analysis of the MRSA isolates.

## Methods and materials

### Study participants

A total of 537 individuals newly diagnosed with HIV infection were recruited at six sites: Amana, Mwananyamala, Temeke Regional Referral, PASADA, Mbagala, and Mnazi Mmoja hospitals in Dar es Salaam, from April 2017 to May 2018, as part of the randomized clinical trial CoTrimResist (ClinicalTrials.gov identifier: NCT03087890).

### Microbiological procedures

#### Specimen collection and bacterial culture

Nasopharynx was swabbed first and, while retracting the swab anterior nares were swabbed. Nasopharyngeal/anterior nares swabs were collected by clinicians and transported to the microbiology laboratory (Muhimbili bacteriology research laboratory) immediately in a cool box at 4 °C. Swabs were cultured on sheep blood agar for isolation of *S. aureus*. Isolates were identified by coagulase, mannitol fermentation and Staphaurex agglutination tests (Remel, Europe Ltd., Dartford, UK).

#### Antimicrobial susceptibility testing and screening for MRSA

Antimicrobial susceptibility testing was performed on Muller Hinton agar, and plates were incubated at 35 °C for 16–18 h. Kirby Bauer disk diffusion method was used for susceptibility testing for penicillin, gentamicin, erythromycin, trimethoprim-sulfamethoxazole, ciprofloxacin, and clindamycin; cefoxitin was also included for initial screening of MRSA (Oxoid, UK). Antimicrobial susceptibility testing was interpreted using Clinical and Laboratory Standards Institute (CLSI) guidelines [13]. The minimum inhibitory concentrations (MICs) for vancomycin and linezolid were determined by E-test (BioMérieux, Marcy-I’Etoile, France).

#### Polymerase chain reaction (PCR) testing

For all cefoxitin-resistant *S. aureus* isolates, we did real-time multiplex PCR targeting the *nuc* and *mecA* genes to confirm the bacterial identity and presence of methicillin resistance, respectively. DNA was extracted by a rapid boiling procedure. PCR was performed using 2× QuantiTect Multiplex PCR NoROX Master Mix (Qiagen), and amplification was carried out on a Light Cycler 480 Instrument II (Roche Diagnostics, Mannheim, Germany). Primers used and PCR conditions have been described previously [14].

#### Whole genome sequencing for MRSA

All 22 MRSA isolates underwent whole genome sequencing (WGS). DNA extraction and whole genome sequencing was performed by MicrobesNG (MicrobesNG, Birmingham, UK). For WGS, Illumina HiSeq technology approach was used (2 × 250 bp paired-end reads protocol) (Illumina, San Diego, CA, USA). Trimming and quality filtering of the sequencing reads were assembled using SPAdes and annotated in GenBank.

Assignment of multilocus sequence typing (MLST) was based on sequencing seven housekeeping genes (*pta*, *arcC*, *tpi*, *aroE*, *gmk*, *yqiL*, and *glpF*). Sequence type (ST) and clonal complex were determined by submission of sequence files to an online MLST database website (https://pubmlst.org/).

For identification of acquired antimicrobial resistance genes, virulence gene, SCCmec and *spa*-type, we used ResFinder v3.2, virulenceFinder 2.0, SCCmecFinder 1.2 and spaTyper 1.0 of the Center for Genomic Epidemiology GEE server (http://www.genomicepidemiology.org/). For analysis of inter-strain whole genome single nucleotide polymorphism (SNPs), we used CSI phylogeny 1.4 (https://cge.cbs.dtu.dk/services/CSIPhylogeny/).

We used Figtree (https://github.com/rambaut/figtree/releases) to construct the phylogenetic tree. The whole genome SNPs tree included 22 ST8 from the present study, *S. aureus* USA300_FPR3757 (accession number CP000255), and 10 already well-characterized ST8 MRSA [15, 16]. This Whole Genome Shotgun project for the present study has been deposited at DDBJ/ENA/GenBank under BioProject number PRJNA649684.

## Results

### Bacterial isolates

In this study, overall nasal/nasopharyngeal carriage rate of *S. aureus* was 14% (77/537), and 29% of the *S. aureus* isolates (*n* = 22) were resistant to cefoxitin, corresponding to a four percent MRSA carrier rate among all participants. All 22 isolates carried the *mecA* and *nuc* gene, confirming methicillin-resistant *S. aureus*. Of 22 isolates, five were isolated from participants who reported having visited an outpatient clinic during the 1 month prior to specimen collection, and one had history of hospitalization during the month prior to specimen collection. Sixteen participants had not visited any outpatient clinics nor been hospitalized recently.

### Antimicrobial susceptibility patterns and genomic resistance traits

All MRSA isolates were susceptible to vancomycin, linezolid, and clindamycin. The overall MRSA antimicrobial susceptibility profiles to other antibiotics are shown in Table 1. Resistance to gentamicin, erythromycin, ciprofloxacin and trimethoprim-sulfamethoxazole and were 95% (21/22), 82% (18/22), 91% (20/22) and 9% (2/22), respectively. Inducible clindamycin resistance was observed in 73% (16/22) of MRSA isolates. Phenotypic susceptibility patterns of all MRSA were highly concordant with genotypic findings (resistome and mutations in genome) (Table 1). All isolates carried *blaZ* gene mediating resistance to penicillin resistance. Resistance to aminoglycosides was mediated by the *aac6’-aph2”* gene. MRSA isolates resistant to erythromycin harbored *ermC* (15/18) and *LmrS* (3/18) resistance genes (Table 1). MRSA resistance to ciprofloxacin was mediated by mutation of the quinolone resistance-determining region (QRDR) sequence in the *gyrA* (S84L) and *parC* (S80Y) genes. All MRSA isolates carried trimethoprim resistance gene *dfrG*, but resistance was not expressed phenotypically (91%, 20/22 susceptible to TMP-SMX). Fosfomycin resistance gene (*FosB*) and tetracycline resistance gene (*tetK*) were detected in 100% (22/22) and 55% (12/22) of MRSA isolates, respectively.

**Table 1:** Phenotypic and genotypic characterization of MRSA

Isolate number	Antimicrobial agents					Antimicrobial resistant genes						QRDRs †mutation		ST	CC	Spa-type	SCCmec type
	Gen	Ery	Sxt	Cip	Cli	β-lactam	Aminoglycoside	Macrolide	Trimethoprim	Tetracycline	Fosfomysin	GyrA	ParC				
4	R	R	S	R	S	*blaZ, mecA*	*aac6’-aph2”*	*ermC*	*dfrG*	*–*	FosB	S84L	S80Y	8	CC8	t1476	IV(2B&5)
6	R	R	S	R	S	*blaZ, mecA*	*aac6’-aph2”*	*ermC*	*dfrG*	*tetK*	FosB	S84L	S80Y	8	CC8	t1476	IV(2B&5)
32	R	R	S	R	S	*blaZ, mecA*	*aac6’-aph2”*	*ermC*	*dfrG*	*–*	FosB	S84L	S80Y	8	CC8	t1476	IV(2B&5)
60	R	S	S	S	S	*blaZ, mecA*	*aac6’-aph2”*	*–*	*dfrG*	*tetK*	FosB			8	CC8	t1476	IV(2B&5)
84	R	R	S	R	S	*blaZ, mecA*	*aac6’-aph2”*	*ermC*	*dfrG*	*–*	FosB	S84L	S80Y	8	CC8	t1476	IV(2B&5)
127	R	R	S	R	S	*blaZ, mecA*	*aac6’-aph2”*	*ermC*	*dfrG*	*–*	FosB	S84L	S80Y	8	CC8	t1476	IV(2B&5)
184	R	R	S	R	S	*blaZ, mecA*	*aac6’-aph2”*	*ermC*	*dfrG*	*–*	FosB	S84L	S80Y	8	CC8	t1476	IV(2B&5)
201	R	R	S	R	S	*blaZ, mecA*	*aac6’-aph2”*	*ermC*	*dfrG*	*tetK*	FosB	S84L	S80Y	8	CC8	t1476	IV(2B&5)
207	R	R	R	R	S	*blaZ, mecA*	*aac6’-aph2”*	*ermC*	*dfrG*	*tetK*	FosB	S84L	S80Y	8	CC8	t1476	IV(2B&5)
228	R	R	S	R	S	*blaZ, mecA*	*aac6’-aph2”*	*ermC*	*dfrG*	*tetK*	FosB	S84L	S80Y	8	CC8	t1476	IV(2B&5)
238	R	R	S	R	S	*blaZ, mecA*	*aac6’-aph2”*	*ermC*	*dfrG*	*–*	FosB	S84L	S80Y	8	CC8	t1476	IV(2B&5)
240	R	R	S	R	S	*blaZ, mecA*	*aac6’-aph2”*	*ermC*	*dfrG*	*tetK*	FosB	S84L	S80Y	8	CC8	t1476	IV(2B&5)
272	R	S	S	R	S	*blaZ, mecA*	*aac6’-aph2”*	*–*	*dfrG*	*–*	FosB	S84L	S80Y	8	CC8	t1476	IV(2B&5)
274	R	R	S	R	S	*blaZ, mecA*	*aac6’-aph2”*	*LmrS*	*dfrG*	*–*	FosB	S84L	S80Y	8	CC8	t1476	IV(2B&5)
370	R	R	S	R	S	*blaZ, mecA*	*aac6’-aph2”*	*ermC*	*dfrG*	*tetK*	FosB	S84L	S80Y	8	CC8	t1476	IV(2B&5)
380	R	R	S	R	S	*blaZ, mecA*	*aac6’-aph2”*	*ermC*	*dfrG*	*tetK*	FosB	S84L	S80Y	8	CC8	t1476	IV(2B&5)
497	R	R	S	R	S	*blaZ, mecA*	*aac6’-aph2”*	*ermC*	*dfrG*	*tetK*	FosB	S84L	S80Y	8	CC8	t1476	IV(2B&5)
2003	R	S	S	R	S	*blaZ, mecA*	*aac6’-aph2”*	*–*	*dfrG*	*–*	FosB	S84L	S80Y	8	CC8	t1476	IV(2B&5)
2010	S	S	R	S	S	*blaZ, mecA*	*–*	*–*	*dfrG*	*–*	FosB			8	CC8	t064	IVa
2028	R	R	S	R	S	*blaZ, mecA*	*aac6’-aph2”*	*LmrS*	*dfrG*	*tetK*	FosB	S84L	S80Y	8	CC8	t1476	IV(2B&5)
2079	R	R	S	R	S	*blaZ, mecA*	*aac6’-aph2”*	*ermC*	*dfrG*	*tetK*	FosB	S84L	S80Y	8	CC8	t1476	IV(2B&5)
2130	R	R	S	R	S	*blaZ, mecA*	*aac6’-aph2”*	*LmrS*	*dfrG*	*tetK*	FosB	S84L	S80Y	8	CC8	t1476	IV(2B&5)

*Pen* penicillin, *Gen* gentamicin, *Ery* erythromycin, *Sxt* trimethoprim-sulfamethoxazole, *Cip* ciprofloxacin, *Cli* clindamycin, *R* resistant, *S* susceptible

†Quinolone resistance-determining regions (QRDR), *ST* sequence type, *CC* clonal complex, *SCCmec* Staphylococcal cassette chromosomes

### Virulence factors and immune evasion cluster genes

Staphylococcal complement inhibitor (*scn*) and Staphylokinase (*sak*), which are among immune evasion cluster (IEC) genes, were detected in all MRSA isolates (Table 2). None of the isolates carried other IEC genes, but one carried staphylococcal enterotoxin A.

**Table 2:** Virulence Genes of MRSA

PID number	Toxin	Exo-enzyme	IEC
4	*lukD, lukE, hlgA, hlgB, hglC, sej, ser*	*aur, splA, splB, slpE*	*sak, scn*
6	*lukD, lukE, hlgA, hlgB, hglC, sej, ser*	*aur, splA, splB, slpE*	*sak, scn*
32	*lukD, lukE, hlgA, hlgB, hglC, sej, ser*	*aur, splA, splB, slpE*	*sak, scn*
60	*lukD, lukE, hlgA, hlgB, hglC*	*aur, splA, splB, slpE*	*sak, scn*
84	*lukD, lukE, hlgA, hlgB, hglC, sej, ser*	*aur, splA, splB, slpE*	*sak, scn*
127	*lukD, lukE, hlgA, hlgB, hglC, sej, ser*	*aur, splA, splB*	*sak, scn*
184	*lukD, lukE, hlgA, hlgB, hglC, sej, ser*	*aur, splA, splB*	*sak, scn*
201	*lukD, lukE, hlgA, hlgB, hglC, sej, ser*	*aur, splA, splB, slpE*	*sak, scn*
207	*lukD, lukE, hlgA, hlgB, hglC, sej, ser*	*aur, splA, splB, slpE*	*sak, scn*
228	*lukD, lukE, hlgA, hlgB, hglC, sej, ser*	*aur, splA, splB, slpE*	*sak, scn*
238	*lukD, lukE, hlgA, hlgB, hglC, sej, ser*	*aur, splA, splB*	*sak, scn*
240	*lukD, lukE, hlgA, hlgB, hglC, sec sej, sel, ser*	*aur, splA, splB*	*sak, scn*
272	*lukD, lukE, hlgA, hlgB, hglC, sej, ser*	*aur, splA, splB*	*sak, scn*
274	*lukD, lukE, hlgA, hlgB, hglC, sej, ser*	*aur, splA, splB, slpE*	*sak, scn*
370	*lukD, lukE, hlgA, hlgB, hglC, sej, ser*	*aur, splA, splB, slpE*	*sak, scn*
380	*lukD, lukE, hlgA, hlgB, hglC, sej, ser*	*aur, splA, splB, slpE*	*sak, scn*
497	*lukD, lukE, hlgA, hlgB, hglC, sej, ser*	*aur, splA, splB, slpE*	*sak, scn*
2003	*lukD, lukE, hlgA, hlgB, hglC, sej, ser*	*aur, splA, splB, slpE*	*sak, scn*
2010	*lukD, lukE, hlgA, hlgB, hglC, sea, seb, sek, seq*	*aur, splA, splB, slpE*	*sak, scn*
2028	*lukD, lukE, hlgA, hlgB, hglC, sej, ser, tst*	*aur, splA, splB, slpE*	*sak, scn*
2079	*lukD, lukE, hlgA, hlgB, hglC, sej, seq, ser*	*aur, splA, splB, slpE*	*sak, scn*
2130	*lukD, lukE, hlgA, hlgB, hglC, sej, ser*	*aur, splA, splB, slpE*	*sak, scn*

*IEC* Immune evasion cluster, *luk* leucocidin, *hlgA* gamma-hemolysin chain II precursor, *hlgB* gamma-hemolysin component B precursor, *hlgC* gamma-hemolysin component C precursor, *sea* enterotoxin, *sea* enterotoxin A *seb* enterotoxin B, *sec* enterotoxin C, *sej* enterotoxin J, *sek* enterotoxin K, *ser* enterotoxin R, *seq* enterotoxin Q, *aur* aureolysin, *spl* serine protease, *sak* Staphylokinase, *scn* Staphylococcal complement inhibitor, *tst* toxic shock syndrome toxin-1

Gamma-hemolysins (*hlgA*, *hlgB*, and *hlgC*) and leucocidin ED genes were detected in all MRSA isolates. Almost all isolates carried staphylococcal enterotoxin genes, with *sej* and *ser* genes being the most predominant. None of the MRSA harbored exfoliative toxin A/B or leucocidin S/F-PV genes. Only one isolates carried toxic shock syndrome toxin 1(*tst*) gene.

### Population genetic structure of MRSA strains

All MRSA isolates were ST8 and belonged to the CC8 lineage (Table 1). All MRSA isolates were genotyped by *spa* type, the most predominant (21/22, 95%) was *spa*-type t1476, and one isolate was *spa*-type t064. On the other hand, genotype ST8-SCC*mec*IV, which is a major CA-MRSA worldwide, were detected in MRSA isolates. All but one of the MRSA isolates belonged to SCC*mec*IV (2B&5) subtype, and the remaining isolate had SCC*mec*IVa subtype.

Overall, genotype ST8-SCC*mec*IV-*spa*-t1476 was the predominant, and all isolates were negative for arginine catabolic mobile element (ACME) type 1 and Panton-Valentine leucocidin (PVL).

### Genetic relatedness among CC8/ST8 MRSA isolates

We compared all 22 CC8/ST8 MRSA isolates from this study with already known ST8 MRSA (USA300) and one MRSA isolate from Tanzania (NZ_FMMT01000000), one from Gabon [16], and eight from the USA [15]. The percentage of reference genome (USA300_FPR3757 (accession number CP000255) covered by all isolates was 91.1%, the size of the reference genome was 2,872,769, and 2,652,029 positions were found in all analyzed genomes. SNP analysis of 22 ST8 MRSA from the present study revealed SNPs differences from 0 to 988, showing high genetic diversity.

**Fig. 1:**
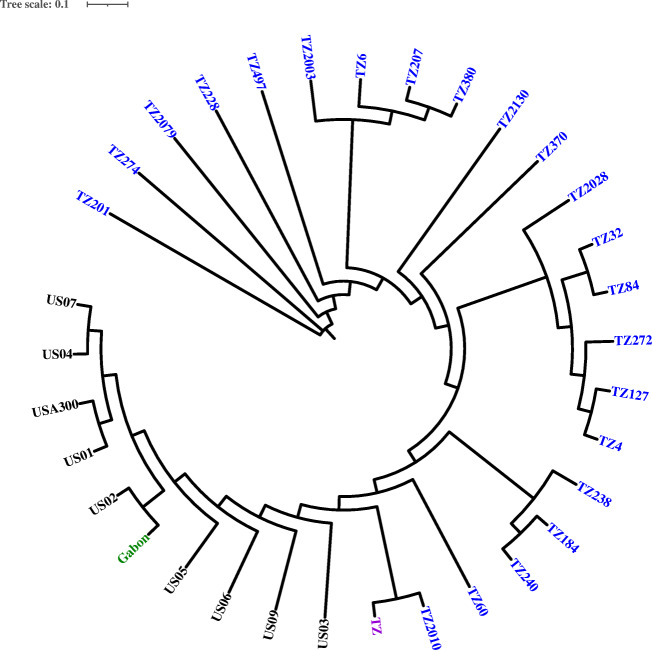
SNP phylogenetic tree for ST8 MRSA isolates. Blue indicates MRSA isolated from this study, pink indicates MRSA isolated previously from Tanzania, black are MRSA isolated from USA at different point of time, and green is an isolate from Gabon

Phylogenetic analysis showed the presence of two distinct clades (Fig. 1). All MRSA isolates with ST8-SCC*mec*IV-*spa*-t1476 from Tanzania formed one clade, had SNPs difference between 1 and 199, and were unrelated to known ST8 clones. Three MRSA isolates had 2 SNPs differences (TZ6, TZ 380, and T207); these were from patients enrolled at different study sites. In one instance, 2 MRSA isolates had 3 SNPs differences (TZ 4 and TZ127). These two instances indicated recent spread of this isolates from a common source.

Two MRSA isolates with pairwise SNP difference of 102 SNPs, both ST8-*spa*-t064 from Tanzania (1 from present study and 1 from previous study), were clustered in another clade with USA300 isolates from the USA and Gabon, Africa. While these two ST8 MRSA isolates from Tanzania possessed SCC*mec*IVa and grouped with ST8 (USA300) from the USA, they were not closely related and had SNPs distances of more than 600 SNPs.

## Discussion

This is the first description of whole genome sequencing data from MRSA isolates from HIV-infected adults in Tanzania and also the first report of nasal/nasopharyngeal MRSA colonization among newly diagnosed HIV-infected adults in Tanzania. Overall, four percent of all participants were nasal/nasopharyngeal MRSA carriers, and all but one belonged to lineage ST8-SCC*mec*IV-t1476. These isolates were collected in the span of 2 years, from six HIV care and treatment centers located in different municipalities in Dar es Salaam. This finding suggests predominance and wide spread of the ST8 CA-MRSA (non-USA300) clone in the HIV-infected population in Dar es Salaam, Tanzania. Diverse lineages of ST8 CA-MRSA dominate different regions in the world. However, the ST8-SCC*mec*IV (USA300) clone has successfully spread in HIV-infected populations in the developed world [17] and is commonly associated with skin and soft tissue infections [18]. No previous study from East Africa has characterized MRSA isolates from HIV-infected population using whole genome sequencing. The only other study in Tanzania, unveiling characteristics of MRSA clinical isolates by whole genome sequencing, found six out of ten MRSA which were ST8 (non-USA), followed by two ST239 and two had unknown sequence type [12]. However, this study did not describe the SCC*mec* and *spa*-type of those isolates.

The ST8-*spa*-t1476 CA-MRSA has rarely been reported outside the African continent, and the types of infection it causes are not known. It has been reported once in the UK in an outbreak of patients with no evidence of links to the African region [19]. The ST8-SCC*mec*V-t1476 clone has been frequently reported among CA-MRSA in the Democratic Republic of Congo (DRC) [20, 21]. Our ST8-SCC*mec*IV MRSA isolates had close resemblance to those circulating in DRC, despite carrying different staphylococcal cassette chromosomes [20, 21]. There is evidence that MSSA acts as reservoir for MRSA before acquisition of staphylococcal cassette chromosomes [22]. Isolates from Tanzania and DRC may have shared common genetic ancestors before each acquiring different SCC*mec*.

On phylogenetic analysis, all ST8-SCC*mec*IV-t1476 from the present study were clustered into one clade and might have shared a common ancestor and were not related to already known ST8 (USA300) clones from the USA and Africa. One isolate from the present study was clustered in a clade with already known ST8 clone, but SNPs analysis showed they were not related. Our findings demonstrate that the epidemiology of the ST8 CA-MRSA clone lineages varies remarkably in different regions of the world. Evolutionary studies have shown that all ST8 clones have a common ancestor but may subsequently acquire certain characteristics like PVL, ACME, and SCC*mec*-type [23]. Repeated introduction of one ST8 CA-MRSA clone in a geographic area may replace existing clones. Our finding and previous data from East and Central Africa confirms predominance of non-USA300 clones [20, 21]. A previous evolutionary study involving ST8 MRSA clones from different continents found that no African ST8 isolates had direct ancestry to the USA300 clades [23]. Further studies are needed to understand the origin and evolution of this clone in our region.

MRSA isolated from newly diagnosed HIV-infected patients were also resistant to gentamicin, erythromycin, and ciprofloxacin but showed low rate of resistance to trimethoprim-sulfamethoxazole phenotypically. However, the presence of the resistance gene *dfrG* in all isolates phenotypically susceptible to trimethoprim-sulfamethoxazole in our study, suggests that this antibiotic could be suboptimal for treatment of MRSA infections. In case it is used, careful clinical monitoring is needed to avoid treatment failure, since in vitro susceptibility might not be matched by susceptibility in vivo.

The present study demonstrates that vancomycin and linezolid could be used for treatment of MRSA infections; however, these drugs are not widely available in East Africa. Macrolides and lincosamides are commonly used in treatment of staphylococcal skin and soft tissue infections. MRSA resistant to macrolides, lincosamides, and streptogramin type B are mainly mediated by *erm*A and *erm*C, which codes for erythromycin ribosomal methylase [24]. We observed predominance of *erm*C from our MRSA isolates (83%), and no isolates carried *erm*A. Previous studies in Tanzania and the DRC reported similar predominance of *erm*C among ST8 CA-MRSA [12, 25]. The observation of 73% inducible clindamycin resistance among MRSA implies that clindamycin may not be a reliable treatment option for infections caused by MRSA. In such cases, treatment with clindamycin may induce clinical resistance in apparently susceptible isolates harboring *erm*C genes [26], leading to clinical treatment failure.

Gentamicin resistance in MRSA was mediated by *aac6’-aph2”* and ciprofloxacin resistance by mutations in the QRDR sequence of the *gyrA* and *parC* genes. This finding is similar to other studies among ST8 CA-MRSA from East Africa and Europe [12, 27, 28]. Although fosfomycin, an older broad spectrum antibiotic [29], has not been commonly used in Tanzania, we still found 100% of MRSA carrying genes conferring resistance to fosfomycin. The high prevalence of FosB in the CA-MRSA isolates is surprising considering the absence of selective pressure by actual use of fosfomycin. This finding could be accounted for by high consumption of other antibiotics including tetracycline which may select for fosfomycin resistance or by the emergence of spontaneous mutation [30, 31].

The ability of MRSA to cause invasive disease depends on virulence factors. Remarkably, the PVL, which is usually present in ST8 CA-MRSA, was lacking from the MRSA isolates in our study. However, several virulence factors were identified including immune evasion cluster, gamma-hemolysins (*hlgA*, *hlgB*, *hlgC*), enterotoxin, and leucocidin ED. Recently, an animal model has shown that leucocidin ED contributes to systemic infection by targeting neutrophils and promoting bacterial growth in vivo [32]. The immune evasion gene cluster enhances capacity of *S. aureus* to colonize, disseminate and persist in a human host [33]. The present study demonstrates that MRSA colonizing the nose/nasopharynx of HIV-infected individuals may have a significant potential to cause invasive disease due to the variety of virulence factors observed.

One caveat of our study is that, although only one of our participants had a history of recent hospitalization and five had visited outpatient clinics, we cannot rule out that MRSA in these patients could have been hospital acquired. However, the fact that all MRSA had the same SCCmecIV type which is commonly found in community-acquired strains supports the notion that the MRSA isolates were community acquired. The anterior nares are a common place of staphylococcal colonization, and swabbing this site is appropriate to detect the bacteria. We swabbed the nasopharynx first, and the anterior nares were swabbed afterwards while retracting the swab. This procedure may have led to falsely low prevalence of *S. aureus*, which could be a limitation of the study.

## Conclusion

ST8 CA-MRSA (non-USA300) was found to be the commonest circulating population structure in newly diagnosed HIV-infected adults in Tanzania. The circulating ST8 CA-MRSA isolates were not related to other common, successful circulating ST8 lineages. The *spa*-type t1476 is predominant in this CA-MRSA. The majority of CA-MRSA was highly resistant to non-beta lactam antibiotics. Screening for colonization of MRSA and intervention in HIV-infected outpatients and inpatients may control the spread of the strain.

## Data Availability

Data are available on request.
